# The impact of chest radiography on patient management in acute trauma care– observation from a level-1 trauma center

**DOI:** 10.1007/s00068-025-02929-0

**Published:** 2025-07-14

**Authors:** Arthur A.R. Sweet, Sophie L. van Wolfswinkel, Tim Kobes, Kim E.M. Benders, Roderick M. Houwert, Luke P.H. Leenen, Pim A. de Jong, Wouter B. Veldhuis, Falco Hietbrink, Mark C.P.M. van Baal

**Affiliations:** 1 Department of Surgery, University Medical Center Utrecht, Utrecht, the Netherlands; 2 Department of Radiology, University Medical Center Utrecht, Utrecht, the Netherlands

**Keywords:** Blunt trauma, Trauma bay, Chest injuries, Chest radiography, Computed tomography

## Abstract

**Purpose:**

This study evaluates the impact of chest radiography on acute interventions in the trauma bay.

**Methods:**

This cross-sectional study was performed on trauma patients admitted to the University Medical Center Utrecht, a level-1 trauma center, during a one-year period. All adult (≥ 16 years) trauma patients who underwent chest radiography in the trauma bay and were subsequently admitted to the hospital were eligible. Patients with non-blunt trauma, initial primary survey in another center, or initial chest radiography obtained outside the shock room were excluded. Patients were categorized as hemodynamically and respiratory compromised or non-compromised patients, and based on symptoms of chest injuries. Descriptive analyses were used.

**Results:**

This study included 780 patients, with a median age of 51 years (IQR 32–68), and 66.2% were male. Comorbidities (ASA 3–4) were seen in 12.8% and the median ISS was 10 (IQR 5–18). There were 382 hemodynamically and respiratory non-compromised patients without symptoms of chest injuries, of whom 255 underwent a subsequent chest CT. No acute interventions were performed in these patients. In symptomatic but hemodynamically and respiratory non-compromised patients (*n* = 289) there were 15 (5.2%) non-urgent chest tube placements prior to CT. Among 109 hemodynamically or respiratory compromised patients there were 16 (14.7%) chest tube placements and five (4.6%) resuscitation surgeries prior to the chest CT.

**Conclusion:**

Omission of chest radiography in hemodynamically and respiratory non-compromised trauma patients presenting in the trauma bay seems safe, provided that a chest CT is already indicated.

## Introduction

Chest trauma is a major cause of mortality as injuries of the chest wall and the intra-thoracic structures can instantly impair circulatory or respiratory functions, and pose a substantial risk of complications [1–3]. The Advanced Trauma Life Support (ATLS) guidelines state that, after all immediate life-threatening chest injuries are identified and treated during the primary survey, chest radiography is indicated to assess for potentially life-threatening chest injuries [4]. Therefore, it has become common practice to obtain chest radiographs in all patients acutely admitted to the trauma bay of the emergency department, regardless of the presence of clinical signs or symptoms of chest injuries. However, studies suggest that chest radiography should be limited to patients with specific clinical indications only [5–7].

Currently, chest computed tomography (CT) is used as the gold standard for diagnosing chest injuries. As chest injuries are often missed in physical examination and are occult on chest radiography, an additional chest CT is indicated to assess these injuries in all patients with a clinical suspicion of any chest injury, and in those who suffered from a high-energy chest trauma [3, 8–10]. An indication for a chest CT reduces the need for chest radiography, provided there is no suspicion of urgent injuries demanding immediate intervention, such as a major pneumothorax or hemothorax, where CT could lead to delay of interventions.

Even though chest radiographs are often routinely obtained in all patients acutely admitted to the trauma bay, we hypothesized that their value is limited in patients who are not hemodynamically or respiratory compromised; especially when a chest CT is already indicated for other reasons. Therefore, this study aimed to evaluate the impact of chest radiography on patient management in the trauma bay, by assessing whether this diagnostic modality leads to immediate interventions before CT imaging is obtained in different patient categories.

## Methods

This cross-sectional study was performed on trauma patients admitted to the University Medical Center Utrecht (UMCU), a level-1 trauma center, between January 2017 and December 2017. Patients were identified using the Dutch National Trauma Registry (DNTR) and the medical health records were reviewed to assess eligibility. Our institutional review board approved a waiver of consent under protocol number 21–481. This study was written according to the Strengthening the Reporting of Observational Studies in Epidemiology (STROBE) statement [11].

### Participants

All adult (≥ 16 years) blunt trauma patients who underwent chest radiography in the trauma bay and who were subsequently admitted to the hospital were eligible. Only the patients who were admitted to the hospital after acute care in the emergency department were included, as only the data of these patients were registered in the DNTR. Patients who died after presentation in the emergency department were also included, while patients who were death on arrival were not included in the DNTR. Patients were excluded if they suffered from other trauma mechanisms than blunt trauma, if they had their initial primary survey in another center and were subsequently transferred to our emergency department, and if they were not admitted to the shock room. All consecutive patients that met the inclusion criteria during a regular year of acute trauma care in our level-1 trauma center were included in this study, aiming to establish a cohort large enough to prevent introducing a selection bias by missing out on any rare cases.

## Standard of care

In our level-1 trauma center, all potentially severely injured patients are admitted with trauma team activation. The trauma team consists of at least a certified trauma surgeon, a certified anesthesiologist, specialized emergency department nurses, a surgery resident, an anesthesiology resident, and radiology technologists. Furthermore, an in-house radiology resident or radiologist is on stand-by to perform a focused assessment with sonography in trauma (FAST) when indicated.

## Chest radiography and CT management

In our trauma center, chest radiographs are routinely taken in the vast majority of patients admitted to the trauma bay, except for evident isolated injuries of the arms or legs. A chest CT was indicated when a patient was admitted with decreased consciousness (e.g., caused by intoxication, neurotrauma, or sedation) and the mechanism of trauma was unknown or was likely to have injured the thorax. In the following cases, a chest CT was indicated without direct suspicion of chest injuries. First, if there were other injuries that indicated the need for a brain and abdominal CT, in which case the thorax was pragmatically included. Second, if other injuries indicated the need for at least three body regions, a total-body CT was performed.

## Explanatory variables

Baseline characteristics obtained from the DNTR were: age, sex, mechanism of trauma, American Society of Anesthesiologists (ASA) classification, maximum Abbreviated Injury Scale (AIS) scores for all body regions [12], Injury Severity Score (ISS) [13], and Glasgow Coma Scale (GCS) score at first arrival of the ambulance at the trauma scene. The following primary survey-related characteristics were obtained from the medical records: airway problems (i.e., obstructed or already intubated), respiratory compromised (i.e., oxygen saturation ≤ 95% with or without oxygen therapy, intubated with an oxygen saturation ≤ 95%), hemodynamically compromised (i.e., systolic blood pressure < 100mmHg, or in case of any hemodynamic support such as noradrenalin, or in case of any blood product administration in the shock room), disability scored using the GCS at first presentation in the shock room. The following signs or symptoms of chest injuries, as reported by the attending trauma surgeon or surgical resident performing the primary survey, were obtained: wounds on the chest, hematomas on the chest, seatbelt signs, asymmetrical chest expansions, decreased breath sounds, subcutaneous emphysema, and pain at the ribs, sternum, clavicles, or thoracic spine.

## Response variables

The primary outcome measure was chest tube placement within the time frame between chest radiography and CT. This intervention was primarily investigated as it directly shows the clinical significance of the prior chest radiography. The lack of urgency, or absence, of immediate interventions based on findings on chest radiography negates the need for prior chest radiography, provided a subsequent CT is already indicated. Secondary outcome measures were findings on the first chest radiograph taken in the trauma bay, as reported by the attending surgical resident performing the primary survey under supervision of the trauma surgeon (i.e., rib fractures, clavicle fractures, pneumothorax, subcutaneous emphysema, deep sulcus signs, hemothorax, lung contusion, mediastinal widening, elevated hemidiaphragm, and whether an endotracheal tube was adequately positioned), and other interventions after chest radiography and before CT (i.e., endotracheal tube pullbacks, and surgical procedures). Additionally, intubation and chest tube placement before chest radiography (i.e., performed by the mobile medical team [MMT] before arrival at the hospital or by the trauma team during the primary survey), and destinations after the emergency department (i.e., CT or resuscitation surgery) were assessed. Management decisions regarding chest radiography, chest tube placement, and CT, were mapped in two decision flowcharts. The first flowchart resembles the practice of a full year of trauma care in our trauma center to clearly illustrate in which patient categories chest radiography might be abundant. The second flowchart shows the suggested patient management for level-1 trauma care based on the findings of this study.

### Statistical analysis

Only descriptive analyses were used in this study, in which explanatory and response variables were presented as means ± standard deviation (SD) for parametric continuous variables, medians with interquartile range (IQR) for non-parametric continuous variables and ordinal variables, and numbers with percentages for categorical variables. The study population was first divided into patients who were hemodynamically or respiratory compromised versus patients who were not. Patients who were not hemodynamically or respiratory compromised, hereafter also referred to as “non-compromised” patients, were further divided into patients with or without signs or symptoms of chest injuries. As this was a descriptive study, no statistical testing was performed.

## Results

### Cohort characteristics

**Fig. 1:**
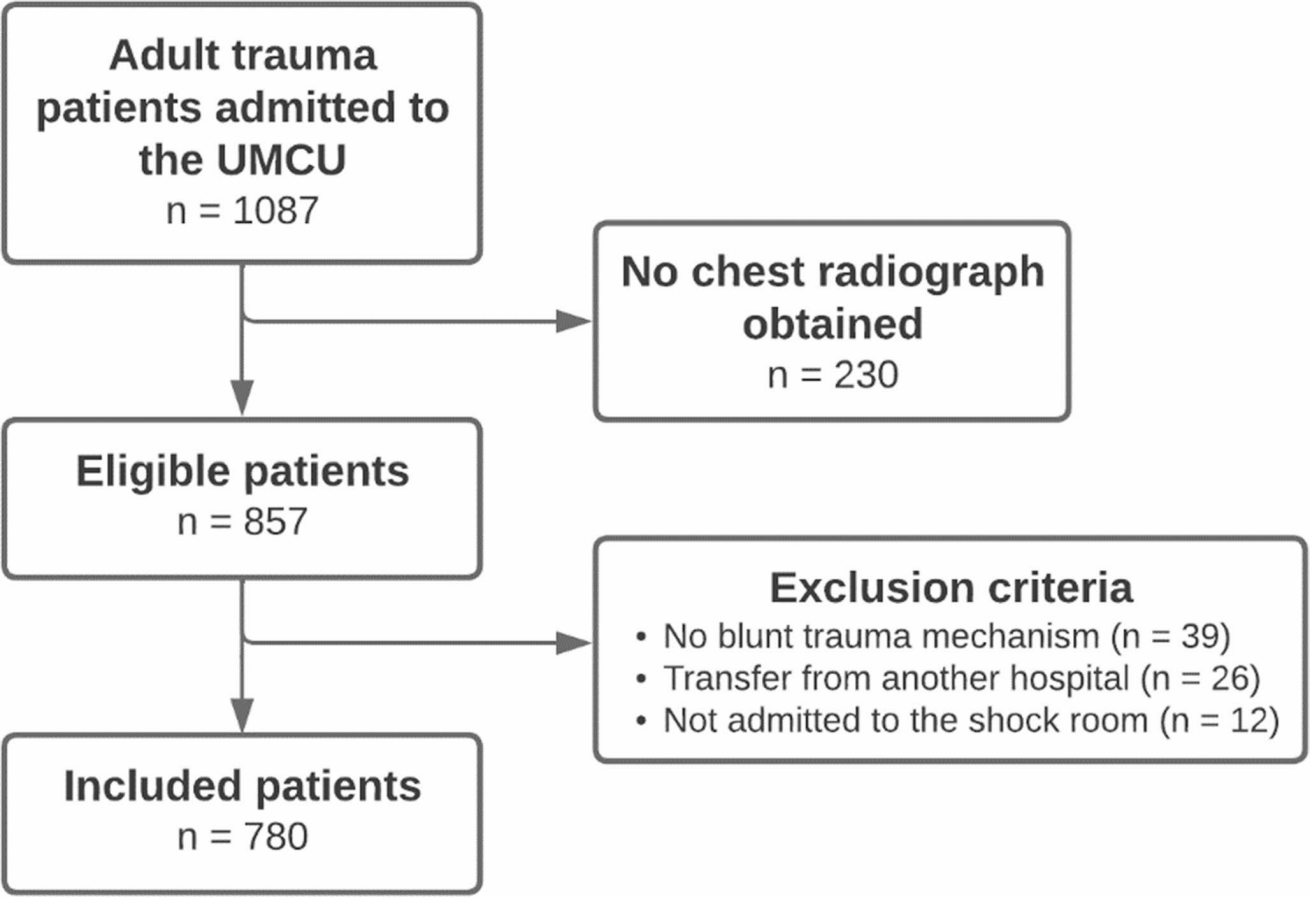
Flowchart of the inclusion and exclusion process. UMCU University Medical Center Utrecht

A total of 1087 patients were acutely admitted for one or more days to the UMCU after a trauma, of whom 857 patients underwent chest radiography (Fig. 1). After excluding 39 patients with other than blunt trauma mechanisms, 26 patients transferred to our trauma bay from another hospital, and 12 patients who were not admitted to the shock room, 780 patients were included. The median age of the total cohort was 51 years (IQR 32–68), and 66.2% were male (Table 1). Comorbidities with an ASA of 3–4 were seen in 12.8%, and the most prevalent mechanisms of trauma were low energy falls (25.5%), bicycle accidents (20.5%), and motor vehicle accidents (18.0%), resulting in a median ISS of 10 (IQR 5–18), and a median GCS score of 15 (IQR 13–15).

**Table 1:** Baseline characteristics

Variable	Total patients
	*n*=780
Age at trauma, median (IQR)	51 (32–68)
Male, n (%)	516 (66.2)
Comorbidity ASA, n (%)	
1–2	680 (87.2)
3–4	100 (12.8)
Mechanism of trauma, n (%)	
Motor vehicle accident	140 (18.0)
Motor cycle accident	90 (11.5)
Bicycle accident	160 (20.5)
Pedestrian	25 (3.2)
Low energy fall	199 (25.5)
High energy fall	108 (13.9)
Other	58 (7.4)
AIS, median (IQR)	
Head	1 (0–3)
Thorax	0 (0–2)
Abdomen	0 (0–0)
Spine	0 (0–2)
Upper extremities	0 (0–1)
Lower extremities	0 (0–1)
ISS, median (IQR)	10 (5–18)
GCS, median (IQR)	15 (13–15)
IQR interquartile range, ASA American Association of Anesthesiologists, AIS abbreviated injury scale, ISS injury severity score, GCS Glasgow Coma Scale	

The majority of the included patients (86%, *n* = 671) were not hemodynamically or respiratory compromised, while 109 (14.0%) patients were compromised in their hemodynamic or respiratory system (Table 2). Clinical signs or symptoms of chest injuries were seen in 42.8% (*n* = 334) of all included patients.

**Table 2:** Findings in primary survey and physical examination of the chest

Variable	Total patients
	*n* = 780
Airway, n (%)	
Obstructed	43 (5.5)
Intubated	83 (10.6)
Respiratory compromised, n (%)	
Oxygen saturation ≤ 95% with oxygen	36 (4.6)
Oxygen saturation ≤ 95% without oxygen	10 (1.3)
Intubated, oxygen saturation ≤ 95%	10 (1.3)
Hemodynamically compromised, n (%)	
Systolic blood pressure < 100 mmHg	30 (3.9)
Hemodynamic support	19 (2.4)
Blood product administration	42 (5.4)
Hemodynamically or respiratory compromised	109 (14.0)
Disability, n (%)	
GCS 8–12	64 (8.2)
GCS < 8	127 (16.3)
Signs and symptoms of chest injuries, n (%)	334 (42.8)
Wounds	16 (2.1)
Hematoma	38 (4.9)
Seatbelt sign	15 (1.9)
Asymmetrical chest expansions	7 (0.9)
Decreased breath sounds	47 (6.0)
Subcutaneous emphysema	11 (1.4)
Pain at the ribs	205 (26.3)
Pain at the sternum	40 (5.1)
Pain at the clavicles	18 (2.3)
Pain at the thoracic spine	80 (10.3)
GCS Glasgow Coma Scale	

### Non-compromised asymptomatic patients

**Fig. 2:**
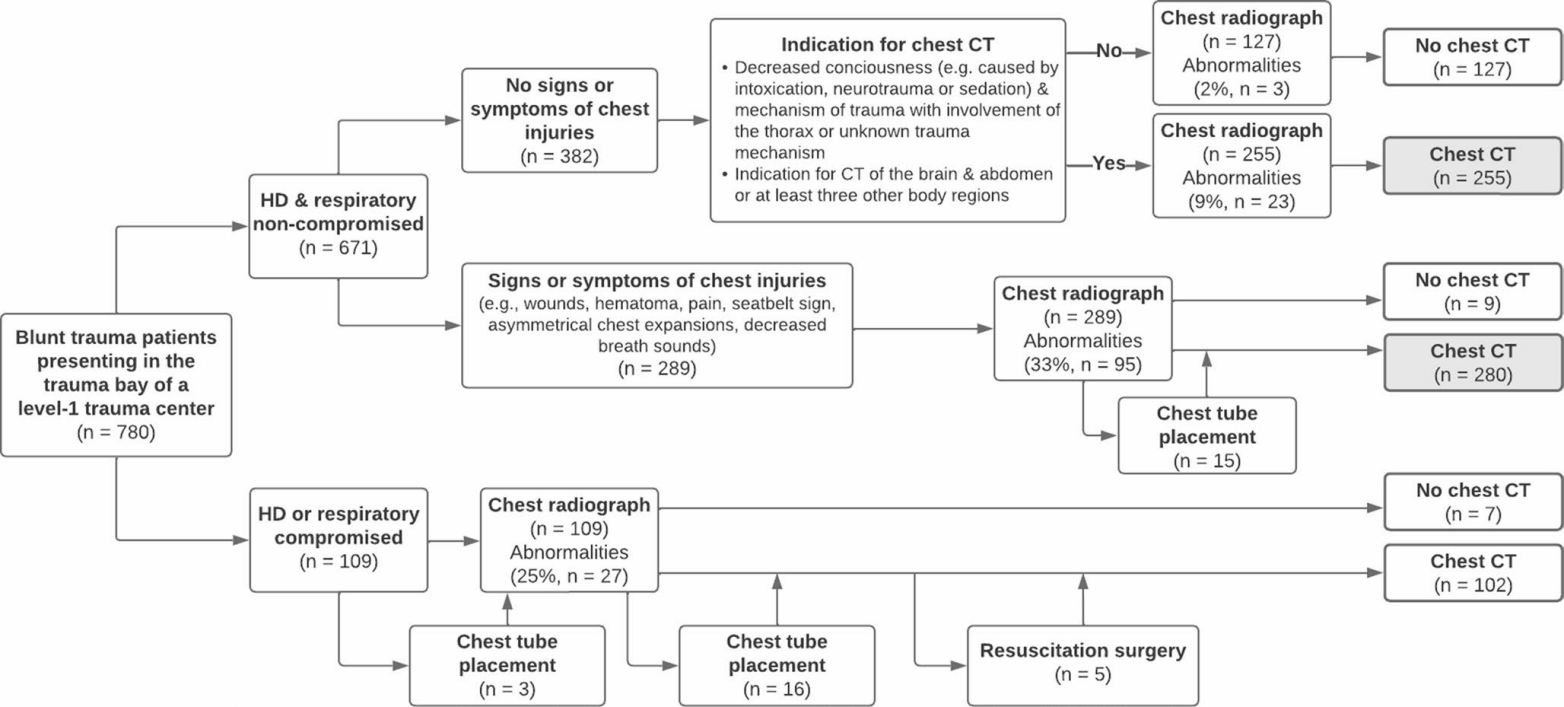
Flowchart of imaging and interventions in acutely admitted blunt trauma patients. The gray boxes indicate the patients in which chest radiographs appear abundant

There were 382 hemodynamically and respiratory non-compromised patients who did not demonstrate any signs or symptoms of chest injuries (Table 3). A total of 61 (16.0%) of these patients were intubated by the MMT during transport or anesthesiologist in the shock room, but no other interventions were performed before chest radiography. In 26 (6.8%) patients, the chest radiograph showed one or more traumatic abnormalities, but these findings did not lead to any acute interventions besides three (0.8%) endotracheal tubes being pulled back. Most of the abnormalities found on the chest radiographs (23 out of 26) were seen in the 255 (66.8%) patients who had an indication for subsequent chest CT (Fig. 2). Three patients had abnormalities on the chest radiograph (i.e., one rib fracture, one clavicle fracture, and one mediastinal widening) that were not further investigated by a following chest CT.

**Table 3:** Findings on chest radiography and interventions

	Total patients	Non-compromised patients		HD or respiratory compromised patients
*Variable*		Asymptomatic	Symptomatic	
	*n* = 780	*n* = 382	*n* = 289	*n* = 109
*Interventions before chest radiograph*,* n (%)*				
Intubation by MMT	79 (10.1)	41 (10.7)	11 (3.8)	27 (24.8)
Intubation in shock room	35 (4.5)	20 (5.2)	1 (0.4)	14 (12.8)
Chest tube placement by MMT	2 (0.3)	0 (0)	1 (0.4)	1 (0.9)
Chest tube placement in shock room	5 (0.6)	0 (0)	2 (0.7)	3 (2.8)
*Findings on chest radiograph by surgeon*,* n (%)*				
Patients with one or more findings	148 (19.0)	26 (6.8)	95 (32.9)	27 (24.8)
Rib fracture(s)	81 (10.4)	6 (1.6)	60 (20.8)	15 (13.8)
Clavicle fracture(s)	31 (4.0)	7 (1.8)	21 (7.3)	3 (2.8)
Pneumothorax				
Unilateral	26 (3.3)	0 (0)	17 (5.9)	9 (8.3)
Bilateral	1 (0.1)	0 (0)	0 (0)	1 (0.9)
Subcutaneous emphysema	12 (1.5)	1 (0.3)	9 (3.1)	2 (1.8)
Deep sulcus sign	1 (0.1)	0 (0)	1 (0.4)	0 (0)
Hemothorax, unilateral	1 (0.1)	0 (0)	1 (0.4)	0 (0)
Lung contusion				
Unilateral	20 (2.6)	6 (1.6)	7 (2.4)	7 (6.4)
Bilateral	3 (0.4)	0 (0)	1 (0.4)	2 (1.8)
Mediastinal widening	20 (2.6)	7 (1.8)	9 (3.1)	4 (3.7)
Elevated hemidiaphragm	6 (0.8)	1 (0.3)	1 (0.4)	4 (3.7)
*Interventions after chest radiograph*,* n (%)*				
Chest tube placement	31 (4.0)	0 (0)	15 (5.2)	16 (14.7)
Indicated by both PE & chest radiograph	20 (2.6)	n/a	12 (4.2)	8 (7.3)
Indicated by PE only	8 (1.0)	n/a	1 (0.3)	7 (6.4)
Indicated by chest radiograph only	3 (0.4)	n/a	2 (0.7)	1 (0.9)
Pull back endotracheal tube	8 (1.0)	3 (0.8)	2 (0.7)	3 (2.8)
Intubation after chest radiograph and before CT	26 (3.3)	10 (2.6)	5 (1.7)	11 (10.1)
*First destination after shock room*,* n (%)*				
CT that included the chest	632 (81.0)	255 (66.8)	280 (96.9)	97 (89.0)
Resuscitation surgery, chest	2 (0.3)	0 (0)	0 (0)	2 (1.8)
Resuscitation surgery, other	3 (0.4)	0 (0)	0 (0)	3 (2.8)
HD hemodynamically, PE physical examination, MMT mobile medical team, CT computed tomography				

### Non-compromised symptomatic patients

A total of 289 hemodynamically and respiratory non-compromised patients demonstrated one or more clinical signs or symptoms of chest injuries (Table 3). Before chest radiography, 12 (4.2%) patients were intubated and three (1.0%) patients underwent chest tube placement. Chest radiography revealed abnormalities in 95 (32.9%) patients, which was followed by 15 (5.2%) chest tube placements and two (0.7%) endotracheal tube pullbacks. Most chest tube placements (12 out of 15) were indicated by a combination of symptoms in physical examination and findings on chest radiography, while one (0.3%) chest tube placement was exclusively indicated by symptoms, and two (0.7%) were exclusively indicated by findings on chest radiography. Five (1.7%) additional patients were intubated following chest radiography. The vast majority (96.9%) of these non-compromised patients directly underwent a chest CT following the chest radiograph. The remaining nine (3.1%) patients all had one minor symptom of chest injuries, such as hematoma or mild pain at the chest, and the attending surgeons decided that a chest CT was not indicated in these patients. There were no further sequalae in the clinical follow-up.

### Compromised patients

There were 109 patients who were hemodynamically or respiratory compromised at arrival in the trauma bay (Table 3). More than a third of these patients (37.6%) were intubated by the MMT before arrival at the hospital or by the anesthesiologist in our trauma bay. One (0.9%) patient underwent chest tube placement by the MMT and three (2.8%) patients underwent chest tube placement by the attending trauma surgeon before chest radiography was performed. Chest radiography showed abnormalities in 27 (24.8%) patients, which was followed by 16 (14.7%) additional chest tube placements. Of these interventions, eight (7.3%) were indicated by symptoms and corresponding findings on radiography, seven (6.4%) were exclusively indicated by symptoms without radiologic abnormalities, and one (0.9%) was indicated by findings on chest radiography without demonstrating any symptoms directly pointing toward intrathoracic pathology. After chest radiography, an additional 11 (10.1%) patients were intubated before transport to the CT or operating room. The vast majority (*n* = 97, 89.0%) underwent a chest CT following chest radiography, while five (4.6%) patients were acutely transported to the operating room for resuscitation surgery without prior CT imaging. Seven (6.4%) patients had isolated injuries of the brain, pelvis, or arms, with no clinical suspicion of chest injuries and, therefore, no chest CT was obtained in these patients.

## Discussion

**Fig. 3:**
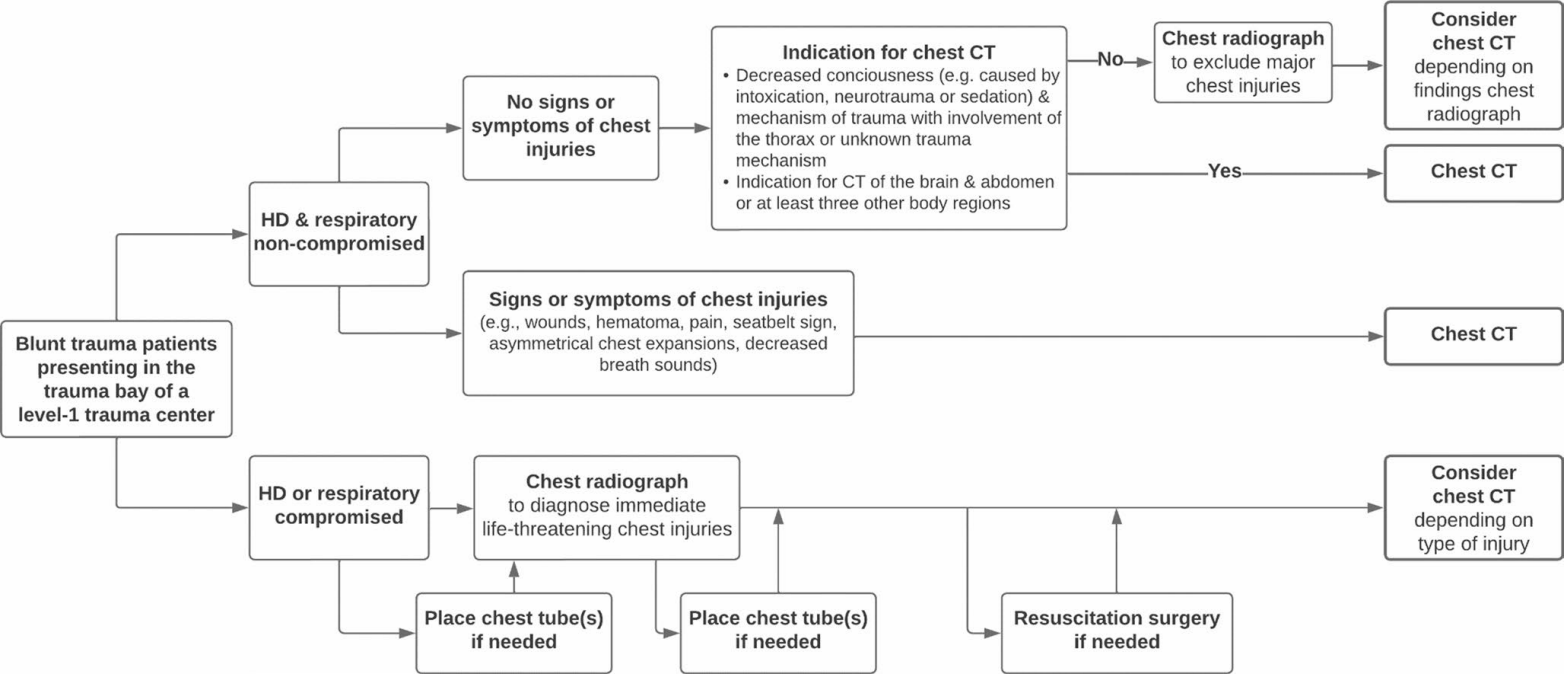
Flowchart of a proposed new chest radiography policy in acutely admitted blunt trauma patients

In this cross-sectional study, the impact of chest radiography on patient management was evaluated in 780 blunt trauma patients acutely admitted to the trauma bay of a level-1 trauma center. We found that in patients who are not hemodynamically or respiratory compromised, chest radiography did not show any injuries requiring acute chest tube placements that would have been missed if the chest radiographs were not taken in these patients. Therefore, we argue that chest radiography can be safely omitted in hemodynamically and respiratory non-compromised trauma patients, provided that a subsequent chest CT is already indicated (Fig. 3). Non-compromised trauma patients with an indication for chest CT comprise more than two third (69.7%) of all included patients, indicating the magnitude of the possible reduction in chest radiography utilization that can be accomplished when implementing a strategy proposed in this study. In hemodynamically or respiratory compromised patients, chest radiography did lead to findings indicating acute interventions before CT, mostly in combination with clinical symptoms.

Acutely admitted but hemodynamically and respiratory non-compromised trauma patients without any specific signs or symptoms of chest injuries often still undergo chest CT, which may be indicated by a decreased consciousness and the trauma mechanism, or as a result of the indication for CT imaging of other body regions with the chest being pragmatically scanned along. This liberal approach for obtaining CT imaging is based on findings in many studies showing that various chest injuries that require non-emergent intervention are frequently missed in physical examination and on chest radiography [8, 9, 14]. In asymptomatic patients with an indication for chest CT investigated in this study, prior chest radiography appeared abundant, as no interventions were performed before the following chest CT was obtained, which is in accordance to other literature [5, 6]. However, if a chest CT is not indicated, it could be argued that chest radiography can still be useful as a quick screening tool.

Non-compromised trauma patients who demonstrate clinical signs or symptoms pointing toward chest injuries demand a chest CT, as a negative chest radiograph cannot rule out occult injuries [15]. In these patients, chest radiography is now used to quickly assess for major chest injuries that require an early intervention before CT. However, as the patients are not compromised in the hemodynamic or respiratory system, the necessity of performing these interventions before CT imaging can be questioned. A third of the chest radiographs in these symptomatic patients showed posttraumatic abnormalities, and 15 (5.2%) chest tubes were placed following radiography, of which 13 patients showed symptoms of a pneumothorax or hemothorax (i.e., decreased breath sounds, asymmetrical chest expansions, or subcutaneous emphysema). Due to the retrospective nature of this study, it remains unknown if these interventions would still have been performed if chest radiography was omitted, and whether this would have been before or after CT. However, as all of these patients subsequently underwent a chest CT, no injuries would have been missed in the end. The medical records of all 15 patients that underwent chest tube placement before CT were reviewed by a staff trauma surgeon to estimate the urgency of acute chest tube placement, concluding that all 15 were non-urgent cases and that a limited time delay in chest tube placement until after the chest CT was deemed safe.

The findings in this study show that the current role of chest radiography in acute trauma care should be reconsidered. Omission of irrelevant chest radiographs is beneficial as it prevents unnecessary radiation exposure, reduces health care costs, and was shown to save an average of three minutes in emergency department length of stay [16]. In modern acute trauma care, with the CT readily available in the emergency department, most patients can skip radiography and directly undergo CT. This leaves two scenarios in which chest radiography can still add value in dedicated level-1 trauma centers; in asymptomatic patients to screen for injuries and thereby prevent the need for a chest CT, and in hemodynamically or respiratory compromised patients to rapidly find out if this is caused by pleural injuries, which can then be immediately treated with chest tube placement or resuscitation surgery before CT imaging is obtained. As the policy on whether to obtain chest radiographs cannot be assessed without taking the policy of the CT into account, our recommendation regarding omission of chest radiography in non-compromised patients predominantly applies to trauma centers with easy access to a CT in or near to the trauma bay, ensuring there is no time delay due to transport.

There were several limitations to this study. First, as this study was performed retrospectively, it remains unknown if the interventions performed in non-compromised patients before CT could have been postponed until after CT. Second, due to our study design and the availability of data, only patients that were admitted to the hospital after acute care in the emergency department were included in this study, introducing a selection bias in which less severely injured patients were left out. Last, we conducted an additional retrospective review of 15 medical records of patients that underwent chest tube placement to assess the urgency of these interventions. As this may have introduced a minor bias, we recommend further investigation of this issue.

In conclusion, omission of chest radiography in non-compromised trauma patients presenting in the trauma bay seems safe, provided that a chest CT is already indicated. Future studies should specifically investigate if non-compromised trauma patients who demonstrate symptoms of chest injuries can await chest tube placement or other treatment until after the results of chest CT.

## Data Availability

Data and code were stored in a secured Research Folder Structure of the UMC Utrecht and are available for future research.
